# Greenhouse gas emissions of self-selected diets in the UK and their association with diet quality: is energy under-reporting a problem?

**DOI:** 10.1186/s12937-018-0338-x

**Published:** 2018-02-21

**Authors:** Kentaro Murakami, M. Barbara E. Livingstone

**Affiliations:** 1 Interfaculty Initiative in Information Studies, University of Tokyo, Tokyo, 113 0033 Japan; 2 Northern Ireland Centre for Food and Health, Ulster University, Coleraine, BT52 1SA UK

**Keywords:** Sustainable diets, Healthy diets, Food consumption, Misreporting

## Abstract

**Background:**

While the admittedly limited number of epidemiological findings on the association between diet-related greenhouse gas emissions (GHGE) and diet quality are not always consistent, potential influence of bias in the estimation of diet-related GHGE caused by misreporting of energy intake (EI) has not been investigated. This cross-sectional study evaluated diet-related GHGE in the UK and their association with diet quality, taking account of EI under-reporting.

**Methods:**

Dietary data used were from the National Diet and Nutrition Survey rolling programme 2008/2009–2013/2014, in which 4-day food diaries were collected from 3502 adults aged ≥19 years. Diet-related GHGE were estimated based on 133 food groups, using GHGE values from various secondary sources. Diet quality was assessed by the healthy diet indicator (HDI), Mediterranean diet score (MDS) and Dietary Approaches to Stop Hypertension (DASH) score. EI misreporting was assessed as reported EI divided by estimated energy requirement (EI:EER).

**Results:**

Mean value of daily GHGE was 5.7 kg carbon dioxide equivalents (CO_2_eq), which is consistent with those reported from a number of national representative samples in other European countries. Mean EI:EER was 0.74. Assuming that all the dietary variables were misreported in proportion to the misreporting of EI, the mean value of the misreporting-adjusted diet-related GHGE was 8.2 kg CO_2_eq/d. In the entire population, after adjustment for potential confounders (i.e., age, sex, ethnicity, socioeconomic classification, smoking status and physical activity), diet-related GHGE were inversely associated with HDI and DASH score but not with MDS. However, with further adjustment for EI:EER, diet-related GHGE showed inverse associations with all three measures of diet quality. Similar associations were observed when only under-reporters (EI:EER < 0.70; *n* = 1578) were analysed. Conversely, in the analysis including only plausible reporters (EI:EER 0.70–1.43; *n* = 1895), diet-related GHGE showed inverse associations with all diet quality measures irrespective of adjustment.

**Conclusions:**

With taking account of EI under-reporting, this study showed inverse associations between diet-related GHGE and diet quality not only in the entire sample but also in the separate analyses of plausible reporters and under-reporters, as well as potential underreporting of diet-related GHGE.

**Electronic supplementary material:**

The online version of this article (10.1186/s12937-018-0338-x) contains supplementary material, which is available to authorized users.

## Background

Rising concerns about climate change led the UK Government to pass the 2008 Climate Change Act, which mandates a reduction in greenhouse gas emissions (GHGE) by 80% by 2050 against the 1990 level [1]. The food supply chain is a major source of GHGE, and the food sector has been estimated to account for 15 to 30% of total GHGE in developed countries [2–4]. Reducing this burden of contemporary food consumption practices on the environment while also improving human health concerns is thus a major challenge of the twenty-first century [5]. While red meat is the top contributor to diet-related GHGE in high-income countries [6–9], higher consumption of red meat has been associated with an increasing risk of total, CVD and cancer mortality [10–12]. Conversely, higher intakes of plant-based foods with lower GHGE such as vegetables, fruits, whole grains and nuts have been associated with lower mortality [11, 13].

Nevertheless, the admittedly limited number of epidemiological findings derived using self-selected diets are not always consistent [14]. Some have reported inverse associations between diet-related GHGE and measures of diet quality [15, 16] while others report positive associations [6]. These heterogeneous results might reflect differences in the types of data sources used, system boundaries in the emission factors adopted, participant characteristics and food and nutrient intake patterns associated with diet-related GHGE [7, 9, 17, 18], in addition to different measures of diet quality. Alternatively, because diet-related GHGE are directly correlated with energy intake (EI) [8, 15, 19], these associations might be confounded by misreporting of EI, particularly under-reporting, which remains an ongoing problem with all self-reported dietary surveys. Although several researchers have raised concerns about the potential influence of this bias in this research field [7, 9, 20, 21], no comprehensive evaluation of its putative impact has yet been made.

A simple and easy way to account for EI under-reporting is to exclude individuals with implausible EI from the analysis, as conducted in several previous studies [7, 9, 20, 21]. However, because EI under-reporting is associated with certain characteristics, particularly overweight and obesity [22–24], this procedure is likely to introduce a selection bias (in addition to reducing sample size) [22, 25]. Another way to account for EI under-reporting is to incorporate the ratio of EI to estimated energy requirement (EER) as a covariate in statistical models [26–29]. Here, we conducted a cross-sectional study to evaluate the GHGE of self-selected diets in the UK and their association with measures of diet quality, while taking account of EI under-reporting.

## Methods

### Data source and analytic sample

The study was conducted under a cross-sectional design using data obtained between 2008/2009 and 2013/2014 as part of the National Diet and Nutrition Survey (NDNS) rolling programme. Details of that study are provided elsewhere [30, 31]. In brief, a nationally representative sample (approximately 500 adults aged ≥19 years and 500 children aged 1.5–18 years) is selected each year via a multi-stage random probability design using a selection of post codes throughout the UK. Further, additional recruitment is carried out in Scotland, Wales and Northern Ireland to obtain country-specific representative data (up to 600 participants per year) [32, 33]. Households are then selected, followed finally by individuals (up to one adult and one child) in participating households. Data collection is conducted throughout the year. The overall response rate ranged from 53% to 56%, depending on survey year [30, 31]. The number of participants aged ≥19 years in the 2008/2009–2013/2014 NDNS rolling programme was 4738. After excluding individuals with missing or invalid information on the variables of interest (*n* = 330 for height and weight; *n* = 1 for smoking status; *n* = 814 for physical activity (PA)) and those without 4-day dietary data (*n* = 91), the final analytic sample comprised 3502 adults. None of the women included in the analysis were pregnant or breastfeeding during the survey period. The NDNS rolling programme is conducted according to the guidelines laid down in the Declaration of Helsinki and all procedures involving human subjects are approved by the Oxfordshire Research Ethics Committee. Written informed consent is obtained from all participants. Data for the present study were obtained from the UK Data Archive.

### Assessment of basic characteristics

Body height (to the nearest 0.1 cm) and weight (to the nearest 0.1 kg) were measured while the participant was wearing light clothes only, without shoes. Body mass index (BMI; kg/m^2^) was calculated as weight (kg) divided by height (m) squared. Self-reported information was collected on the following variables. Socioeconomic status was determined by the National Statistics socioeconomic classification [34], and categorized as higher and managerial occupation, intermediate occupation, routine and manual occupation or other. Ethnicity was categorized as white or nonwhite. Smoking status was categorized as current, former or never smoker.

PA was assessed by a validated questionnaire (i.e., Recent Physical Activity Questionnaire) [35]. In brief, this questionnaire is designed to assess a wide range of PA in the past month in four domains (home, work, commuting and leisure activities). As described elsewhere [36], the time per day spent in moderate-to-vigorous physical activity (MVPA) was calculated. Descriptions on PA categories (i.e., sedentary, low active, active and very active) and the amount of time spent in MVPA are available in the US Dietary Reference Intakes (DRI) [37]. Specifically, the guidelines state that participation in an additional 30 min of moderate activity raises an individual from the sedentary to the low-active PA category, and about 60 min of moderate activity raises an individual from the sedentary to the active PA category [38, 39]. Thus, the following four categories on PA were created and used in the present analysis: sedentary (< 30 min of MVPA), low active (≥30 to < 60 min of MPVA), active (≥60 to < 120 min of MVPA) and very active (≥120 min of MVPA).

### Dietary assessment

Dietary data were collected using a 4-consecutive-day estimated food diary. A detailed description of the procedure has been published elsewhere [40]. In brief, the participants were requested to maintain a record of all items eaten or drunk for four consecutive days. The recording schedule was designed so that all days of the week were evenly represented. Each participant was supplied with a recording diary and a set of photographs of 15 major foods in small, median and large portion sizes, as well as written and verbal instructions by trained interviewers on how the diary should be maintained. The participants used the photographs to describe the portion sizes of foods they consumed. For other foods, portion sizes were described in household measures (e.g., one tablespoon of baked beans) or as the weight indicated on the food package. Trained interviewers visited the household once during the recording period and once within three days after it to check on the completeness of food recording, and whenever necessary sought additional information or modification of the record. The collected diaries were checked by trained coders and editors for coding of food items and for portion sizes. Estimates of daily intake for foods, energy and selected nutrients were calculated based on the Department of Health’s NDNS nutrient data bank, which itself is based on McCance and Widdowson’s Composition of Foods series [41]. For all dietary variables, mean values over the 4 recording days were used in the analysis.

### Assessment of diet quality

Overall diet quality was assessed using the healthy diet indicator (HDI) [42, 43], Mediterranean diet score (MDS) [43, 44] and Dietary Approaches to Stop Hypertension (DASH) score [45, 46] (see Additional file 1: Table S1). These diet quality measures have been prospectively associated with certain desired health outcomes, including lower mortality [16, 42, 44, 47, 48]. The HDI was chosen as a diet quality measure for its primary focus on nutrient intake, while the MDS and DASH score were selected for their primary focus on intake of food group/type.

The HDI incorporates six nutrients and one food group (saturated fat; polyunsaturated fat; protein; dietary fibre; cholesterol; and non-milk extrinsic sugar; and fruits and vegetables) [42, 43]. If intake remained within the recommended WHO guideline range, that component was assigned a score of one; if not, it was assigned a score of zero, giving a total score range of zero to seven, with a higher score reflecting a healthier dietary pattern.

The MDS represents the Mediterranean diet type, based on the consumption of nine components (vegetables; legumes; fruits, nuts and seeds; cereals; fish; unsaturated to saturated fat ratio; dairy products; meat; and alcohol) [43, 44]. Regarding alcohol, moderate intake (i.e., 10–50 g/d for men and 5–25 g/d for women) was assigned a score of one. For dairy products and meat, a score of one was assigned to intake below or equal to the sex-specific median. For other components, a score of one was assigned to intake above or equal to the sex-specific median. Scores for the nine components were summed to give a total possible range of zero to nine, with a higher score reflecting better consistency with a Mediterranean-type diet.

The Fung’s DASH score, originally developed for the US Nurse’s Health Study [45], was used with the slight modification of Penney et al., which considered UK dietary habits [46]. Components considered in this measure are vegetables, fruits, whole grain foods, nuts and legumes, low fat dairy products, red and processed meats, non-milk extrinsic sugar, and sodium. Participants were classified for each component into sex-specific quintiles by intake. Scores ranged from 1 to 5 for each quintile; for vegetables, fruits, whole grain foods, nuts and legumes, and low fat dairy products, higher intakes were given higher scores, while for red and processed meats, non-milk extrinsic sugar and sodium, higher intakes were given lower scores. Scores for the eight components were summed, giving a total possible score range of 8, indicating lowest adherence, to 40, for maximum adherence.

### Estimation of diet-related greenhouse gas emissions

Diet-related GHGE, expressed as kg of carbon dioxide equivalent (CO_2_eq), were calculated as the sum of the product of GHGE value of food group and intake of food group. GHGE values of each food group were taken from Green et al. [49], where values were calculated using life cycle analysis data from the literature, mainly from the UK and Europe, based on all stages from food production, packing, distribution, storage/refrigeration, transportation (farm-to-outlet and retailer-to-home), food handling/preparation (including trimmings and cooking losses) to consumer waste (including spoilage and plate waste). The GHGE value of each food group (*n* = 133) used in this study is shown in Additional file 1: Table S2. GHGE for the following four food groups were not included in the analysis because of a lack of information in Green et al. [49]: tap water only; beverages dry weight; nutrition powders and drinks; and savoury sauces pickles gravies and condiments.

### Evaluation of the accuracy of energy intake misreporting

Misreporting of EI was evaluated based on the ratio of EI to EER, namely, the procedure proposed by Huang et al. [50]. Participants were identified as plausible reporters, under-reporters or over-reporters of EI according to whether the individual’s ratio was within, below or above the 95% confidence limits of the expected EI:EER of 1.0. We calculated each subject’s EER, based on the information on age, weight, height and PA category (i.e., sedentary, low-active, active or very active, as mentioned above), with the use of equations published from the US DRI [37]. The sex- and age-specific equations for use in populations with a range of weight statuses [37] were used. The 95% confidence limits of the expected EI:EER ratio of 0 on the natural log scale were calculated, taking into account CV in intakes and other components of energy balance (i.e., the within-subject variation in EI: 23%; the error in the EER equations: 11%; and the day-to-day variation in total energy expenditure: 8.2%) [37, 50, 51] as well as the number of diet recording days (4 d). Consequently, under-reporters, plausible reporters and over-reporters were defined as having an EI:EER < 0.70, 0.70–1.43 and > 1.43, respectively.

### Statistical analysis

Statistical analyses were performed using SAS statistical software (version 9.4, SAS Institute). All reported *P* values are two-tailed, and *P* < 0·05 was considered to be statistically significant. Initially, analyses were conducted separately for men and women, but the results were essentially the same, albeit that mean value of diet-related GHGE and the percentage of under-reporters were higher in men than women, as shown in the Results section. We therefore present the results for men and women combined.

Descriptive data are presented as means and SD for continuous variables and percentages of participants for categorical variables. Differences in diet-related GHGE across categories of each of the selected characteristics were examined by the independent *t* test or ANOVA. When the overall *P* value from ANOVA was < 0.05, Bonferroni’s post hoc test was performed. Differences in plausible reporters and under-reporters (but not over-reporters, because of their small number) were tested by the independent *t* test for continuous variables and the chi-square test for categorical variables. Correlations of diet-related GHGE and measures of diet quality (HDI, MDS and DASH score) with EI and EI:EER were investigated using Pearson correlation analyses.

Associations between diet-related GHGE and measures of diet quality were investigated by linear regression analyses using the PROC REG procedure. Potential confounding factors considered were age, sex, ethnicity, socioeconomic classification, smoking status and physical activity (model 1). Further adjustment was made for EI:EER (model 2). All analyses were conducted for the entire population, and also for plausible reporters only and for under-reporters only. It should be noted that adjustment for EI:EER in the analysis of plausible reporters was made because they are just those whose EI values are not extreme enough to be labelled as under- or over-reporters, and thus relatively small misreporting may still occur in plausible reporters.

Data used in the present analysis were weighted to account for the survey’s complex sampling structure and non-response bias, using the published weights for combining data from the NDNS rolling programme 2008/2009 to 2013/2014, which has been described elsewhere [33, 52].

## Results

This analysis included 1429 men and 2073 women with a mean age of 48 years (Table 1). The mean value of crude diet-related GHGE was 5.7 kg CO_2_eq/d (1st, 5th, 95th and 99th percentiles: 2.1, 3.0, 9.5 and 11.6 kg CO_2_eq/d, respectively). Compared with EER, EI was under-reported by an average of 26%. Assuming that all the dietary variables were misreported in proportion to the misreporting of EI, the mean value of the misreporting-adjusted diet-related GHGE was 8.2 kg CO_2_eq/d (1st, 5th, 95th and 99th percentiles: 3.4, 4.2, 14.1 and 17.0 kg CO_2_eq/d, respectively). There were significant associations between diet-related GHGE and all of the potential confounding factors considered (Additional file 1: Table S3).

**Table 1:** Characteristics of participants ^a^

	All (*n* = 3502) ^b^		Plausible reporters (*n* = 1895)		Under-reporters (*n* = 1578)		
	Mean	SD	Mean	SD	Mean	SD	*P* ^c^
Age (years)	47.6	17.7	49.9	18.4	45.0	16.2	< 0.0001
Sex (% male)	49.2		44.3		55.3		< 0.0001
Ethnicity (% white)	89.4		90.0		88.4		0.13
Socioeconomic classification (%)							< 0.0001
Higher and managerial occupation	44.3		46.9		41.4		
Intermediate occupation	19.7		20.4		18.7		
Routine and manual occupation	31.7		27.9		36.1		
Other	4.4		4.8		3.9		
Smoking status (%)							< 0.0001
Current	21.5		17.9		24.9		
Former	23.9		23.6		24.6		
Never	54.7		58.5		50.5		
Physical activity (%)							< 0.0001
Sedentary	39.1		50.4		25.0		
Low active	19.4		21.9		16.8		
Active	19.6		16.9		23.1		
Very active	21.8		10.7		35.1		
BMI (kg/m^2^)	27.4	5.4	26.4	4.9	28.6	5.6	< 0.0001
EI (kJ/d)	7653	2375	8611	2119	6410	1847	< 0.0001
EER (kJ/d)	10,699	2673	9788	2340	11,821	2803	< 0.0001
EI:EER	0.74	0.23	0.89	0.14	0.55	0.11	< 0.0001
Diet-related GHGE (kg CO_2_eq/d)	5.7	2.1	6.3	2.1	5.0	1.8	< 0.0001
HDI	2.3	1.1	2.2	1.2	2.4	1.1	< 0.0001
MDS	4.5	1.7	4.6	1.7	4.3	1.7	< 0.0001
DASH score	24.3	5.2	23.9	5.2	24.7	5.2	< 0.0001

*BMI* body mass index, *CO*_*2*_*eq* carbon dioxide equivalents, *DASH* Dietary Approaches to Stop Hypertension, *EER* estimated energy requirement, *EI* energy intake, *GHGE* greenhouse gas emissions, *HDI* healthy diet indicator, *MDS* Mediterranean diet score

^a^Plausible reporters were defined as participants with an EI:EER 0.70–1.43; under-reporters were defined as participants with an EI:EER < 0.70

^b^ Including over-reporters (*n* = 29), defined as participants with an EI:EER > 1.43

^c^ *P* values for differences between plausible reporters and under-reporters based on the independent *t* test for continuous variables and the chi-square test for categorical variables

The percentages of plausible reporters and under-reporters of EI were 54 and 45%, respectively (only 29 participants (0.8%) were classified as over-reporters) (Table 1). Compared with plausible reporters, under-reporters were more likely to be younger, male, employed in routine and manual occupations, current smokers and physically active. They also had higher means of BMI, EER, HDI and DASH score and lower means of diet-related GHGE, EI and MDS.

In the entire population, red meat contributed about one-quarter of diet-related GHGE (24.4%), followed by dairy products, which contributed about one-eighth (13.6%) (Table 2). Other important contributors (≥5%) were soft drinks (7.3%), cereals (6.9%), sugar and confectioneries (6.0%), fish (6.0%), white meat (5.9%), fat and oils (5.5%) and vegetables (5.3%). Similar results were observed in the analysis of plausible reporters only and of under-reporters only.

**Table 2:** Food group intake and percentage contribution of each food group to diet-related GHGE ^a^

	All (*n* = 3502) ^b^				Plausible reporters (*n* = 1895)				Under-reporters (*n* = 1578)			
	Intake (g/d)		Contribution to GHGE (%)		Intake (g/d)		Contribution to GHGE (%)		Intake (g/d)		Contribution to GHGE (%)	
	Mean	SD	Mean	SD	Mean	SD	Mean	SD	Mean	SD	Mean	SD
Red meat	85.2	67.1	24.4	16.9	90.5	69.8	24.2	16.2	77.9	61.3	24.8	17.8
White meat	44.3	55.4	5.9	6.8	44.9	59.3	5.4	6.3	42.6	47.9	6.5	7.3
Fish	30.8	37.3	6.0	7.5	35.1	39.3	6.4	7.4	25.5	33.9	5.5	7.5
Dairy products	223.0	150.3	13.6	8.6	247.6	155.4	14.3	8.7	191.8	134.6	12.7	8.4
Eggs	20.5	33.9	1.8	2.5	22.9	40.5	1.8	2.5	17.4	23.3	1.8	2.5
Cereals	185.5	96.8	6.9	5.1	198.8	99.9	6.8	4.9	168.9	89.3	7.1	5.3
Potatoes	90.1	67.1	2.8	2.2	96.6	68.8	2.8	2.0	82.2	64.3	2.9	2.4
Vegetables	139.2	93.3	5.3	4.0	148.3	91.2	5.1	3.6	127.8	94.3	5.5	4.5
Beans and pulses	24.4	35.8	0.7	1.2	25.4	35.7	0.7	1.0	23.2	36.1	0.8	1.5
Nuts and seeds	3.6	11.6	0.2	0.6	4.4	11.6	0.2	0.4	2.8	11.6	0.1	0.7
Fruit	101.2	105.5	2.8	3.0	111.3	108.5	2.8	2.8	88.8	99.4	2.8	3.2
Fats and oils	13.8	11.1	5.5	4.9	16.0	11.6	5.8	5.0	11.0	9.1	5.0	4.6
Sugar and confectioneries	97.7	78.4	6.0	5.8	112.7	78.3	6.2	5.2	77.1	70.2	5.6	6.4
Soft drinks	222.6	347.1	7.3	10.0	223.6	354.4	6.6	8.9	216.4	322.0	8.1	10.9
Fruit juice	50.1	101.9	2.1	4.0	59.6	110.5	2.3	4.0	37.4	78.6	1.8	3.8
Alcoholic beverages	212.2	401.5	4.8	8.0	252.2	452.0	5.2	8.0	165.0	318.8	4.4	8.0
Tea, coffee and water	783.8	494.3	3.9	4.5	828.3	492.2	3.5	3.7	733.0	492.6	4.5	5.3

*GHGE* greenhouse gas emissions

^a^ Plausible reporters were defined as participants with a ratio of reported energy intake (EI) to estimated energy requirement (EER) 0.70–1.43; under-reporters were defined as participants with an EI:EER < 0.70

^b^ Including over-reporters (*n* = 29), defined as participants with an EI:EER > 1.43

Diet-related GHGE were strongly positively correlated with both EI and EI:EER in the entire population (Additional file 1: Table S4). While HDI and DASH score showed weak inverse correlations with EI and EI:EER, MDS was weakly and positively correlated with these measures. The correlations became somewhat weak (or nonsignificant) when analysed for plausible reporters and under-reporters separately, except for inverse correlations for HDI and positive correlations for MDS in under-reporters.

Table 3 shows associations between diet-related GHGE and diet quality measures. In the entire population, after adjustment for potential confounding factors (i.e., age, sex, ethnicity, socioeconomic classification, smoking status and physical activity; model 1), diet-related GHGE were inversely associated with HDI and DASH score but not with MDS. However, with further adjustment for EI:EER (model 2), diet-related GHGE showed inverse associations with all three measures of diet quality. Similar associations were observed when only under-reporters were analysed (albeit that the inverse association for MDS did not reach statistical significance). Conversely, in the analysis including only plausible reporters, diet-related GHGE showed inverse associations with all diet quality measures irrespective of adjustment.

**Table 3:** Associations between diet-related GHGE and diet quality measures ^a^

	Model 1 ^b^			Model 2 ^c^		
	β ^d^	SE ^d^	*P*	β ^d^	SE ^d^	*P*
All (*n* = 3502) ^e^						
HDI	−0.12	0.01	< 0.0001	−0.14	0.01	< 0.0001
MDS	0.002	0.01	0.86	−0.07	0.02	< 0.0001
DASH score	−0.51	0.04	< 0.0001	−0.42	0.05	< 0.0001
Plausible reporters (*n* = 1895)						
HDI	−0.12	0.01	< 0.0001	−0.14	0.02	< 0.0001
MDS	−0.08	0.02	0.0003	−0.10	0.02	< 0.0001
DASH score	−0.51	0.06	< 0.0001	−0.47	0.06	< 0.0001
Under-reporters (*n* = 1578)						
HDI	−0.15	0.02	< 0.0001	−0.15	0.02	< 0.0001
MDS	0.01	0.03	0.70	−0.05	0.03	0.06
DASH score	−0.42	0.07	< 0.0001	−0.35	0.08	< 0.0001

*DASH* Dietary Approaches to Stop Hypertension, *EER* estimated energy requirement, *EI* energy intake, *GHGE* greenhouse gas emissions, *HDI* healthy diet indicator, *MDS* Mediterranean diet score

^a^ Plausible reporters were defined as participants with an EI:EER 0.70–1.43; under-reporters were defined as participants with an EI:EER < 0.70

^b^ Adjustment was made for age (years, continuous), sex (male or female), ethnicity (white or nonwhite), socioeconomic classification (higher and managerial occupation, intermediate occupation, routine and manual occupation or other), smoking status (current, former or never), and physical activity (sedentary, low active, active or very active)

^c^ Adjustment was made for variables used in model 1 and EI:EER (continuous)

^d^ Indicating the change of diet quality measures with a 1-kg of carbon dioxide equivalents increase of diet-related GHGE (per day)

^e^ Including over-reporters (*n* = 29), defined as participants with an EI:EER > 1.43

## Discussion

To our knowledge, this is the first study to examine diet-related GHGE in relation to measures of diet quality, taking account of EI under-reporting. Assuming that all the dietary variables were misreported in proportion to the misreporting of EI, diet-related GHGE were underestimated by 30% in this cross-sectional study based on the UK NDNS rolling programme. In the entire population, diet-related GHGE were inversely associated with HDI and DASH score but not with MDS after adjustment for potential confounders. However, with further adjustment for EI:EER, diet-related GHGE showed inverse associations with all three diet quality measures. Similar associations were observed when only under-reporters were analysed, while in the analysis including only plausible reporters, diet-related GHGE showed inverse associations with all diet quality measures irrespective of adjustment. These findings highlight the importance of taking account of EI misreporting in the entire sample, rather than just excluding EI misreporters.

Our mean estimate of diet-related GHGE was 5.7 kg CO_2_eq/d, which is consistent with those reported from a number of national representative samples in other European countries, such as France (4.1 kg CO_2_eq/d) [6], Ireland (6.5 kg CO_2_eq/d) [9], Sweden (women: 4.1 kg CO_2_eq/d; men: 5.5 kg CO_2_eq/d) [18] and the Netherlands (women: 3.7 kg CO_2_eq/d; men: 4.8 kg CO_2_eq/d) [7]. These differences may be due to differences in the types of data sources used and in system boundaries in the emission factors adopted, in addition to differences in dietary assessment methods and participant characteristics (such as age range and dietary habits). However, we observed that the mean value of diet-related GHGE increased by 30% (8.2 kg CO_2_eq/d) with an assumption that all the dietary variables were misreported in proportion to the misreporting of EI. Under-reporting of EI was on average 26% in this study, which is quite similar to that in a subsample of the NDNS rolling programme as assessed against total energy expenditure by the doubly labelled water method (34% in individuals aged 16–64 years and 29% in those aged ≥65 years) [30]. Indeed, this degree of under-reporting is quite common in dietary surveys [53]. It is therefore highly likely that diet-related GHGE will be underestimated to a certain degree if EI misreporting is not taken into account.

In this study, red meat was the top contributor to diet-related GHGE, followed by dairy products. This is consistent with previous studies in Ireland [9], the Netherlands [7] and France [8]. Thus, this study provides further evidence based on self-selected diet that reducing meat consumption is likely to contribute to lower diet-related GHGE [2], as indicated in modeling studies [49, 54, 55]. Nevertheless, avoidance or lower intake of animal foods such as red meat may also contribute to nutritional inadequacy of several micronutrients such as iron, zinc and vitamin B-12 [56]. In any case, further research is required to determine the amount of meat consumption (particularly red meat) which is optimum for not only human health but also for the health of the planet.

Misreporting of dietary intake is a common phenomenon. It appears to arise non-randomly [24, 53] and be selective for different kinds of foods, and accordingly nutrients [57–60]. Misreporting has the potential to cause differential errors in dietary data, which in turn hampers the interpretation of studies concerning diet and health. In the worst case it can lead to spurious diet-health relationships [22, 25, 58]. In our present study, EI misreporter (i.e., under-reporters and over-reporters) prevalence was 46%, which is similar to that in previous studies based on similar dietary assessment methods (37% [61] and 38% [62]). The present findings are similarly consistent with many other studies [22–24, 53, 61] showing that under-reporters differ considerably from plausible reporters, such as with regard to higher body fatness, higher PA, lower socio-economic status and rate of current tobacco use. These differences notwithstanding, the associations of diet-related GHGE with diet quality (in addition to the contribution of food groups to GHGE) seen in under-reporters were reasonably similar to those seen in plausible reporters, after taking into account EI:EER. Nevertheless, misreporting (particularly under-reporting) of EI appeared to confound the inverse associations with diet quality (i.e., MDS), suggesting the importance of taking into account of EI misreporting. The reason for this observation is not precisely known, but one speculation is that even though foods are differentially misreported, these reporting errors are correlated with EI misreporting, to some extent at least (Pearson correlations between EI and food group intakes ranged from 0.08 to 0.46 in this study). This does not conflict with observations based on biomarkers of protein, potassium and sodium (24-h urinary excretion) [57, 60, 63].

After taking account of EI misreporting, we identified inverse associations between diet-related GHGE and all three measures of diet quality (i.e., HDI, MDS and DASH score). An inverse association with DASH score was similarly observed in British [15] and Dutch [16] populations. An inverse association with HDI was also observed in the Dutch study [16]. Conversely, a study in France showed a positive association between diet-related GHGE and diet quality assessed as a composite measure of three different indices, namely mean adequacy ratio, mean excess ratio and energy density [6]. These heterogeneous findings may be at least partly explained by the use of different measures for diet quality, given that different diet scores have conceptual differences. Given the improbability of identifying a diet quality index which is able to capture all aspects of healthy diets, the use of different measures of diet quality, as conducted in this study, may be an important approach to ensuring the robustness of the findings.

The strengths of this study include its detailed dietary information obtained from a 4-d food diary, measured anthropometric data, and use of an individualized measure of EER to assess EI misreporting in a representative sample in the UK. However, several limitations also warrant mention. First, because of the limited availability of food-level GHGE data, we obtained the estimates of diet-related GHGE from GHGE values for 133 food groups, rather than those assigned to individual food items (> 2000 in the NDNS). Accordingly, we have likely underestimated variation in diet-related GHGE. Nevertheless, this strategy is currently the only feasible approach in many epidemiological studies given the challenges of building a standardised comprehensive food database [64]. Further, our estimate of diet-related GHGE is of a comparable magnitude to those provided in studies in Europe [6, 7, 9, 18]. Second, environmental impact was indicated using GHGE only, rather than such criteria as land and water use or biodiversity. As highlighted in a recent systematic review [65], these should be simultaneously taken into consideration in future studies.

At present, the use of doubly labelled water as a biomarker is the only way to secure unbiased information on energy requirements in free-living settings [53]. Nevertheless, this technique is expensive and cannot be practically applied to large-scale dietary surveys like the NDNS. As a replacement, we determined EER with the use of published equations [37]. In the absence of measured total energy expenditure, these equations with high *R*^2^ values (0.82 for men and 0.79 for women) [37] would serve as the best proxy, although the selection of PA category was based on self-report (i.e. a validated questionnaire), which may be susceptible to reporting bias. This notion is evidenced, at the population level at least, by the finding of under-reporting in this study, and of under-reporting observed in comparison with total energy expenditure using doubly labelled water in a subsample of the NDNS rolling programme [30], as mentioned above.

Another limitation of this study is its relatively low response rate (53 to 56% among survey years). However, an analysis based on the previous NDNS, which had a response rate for dietary recording of 47%, concluded that there was no evidence to suggest confounding by a serious non-response bias in the NDNS [66]. Finally, although we adjusted for a variety of potential confounding variables, residual confounding could not be ruled out.

## Conclusion

This cross-sectional study, based on self-selected diets in the UK, revealed that consistent inverse associations between diet-related GHGE and measures of diet quality were observed when misreporting of EI was taken into account, as well as potential underestimation of diet-related GHGE. Thus, misreporting (particularly under-reporting) of EI appeared to confound the inverse associations with diet quality. However, because these associations were observed not only in the entire population but also in both plausible reporters and under-reporters separately, simple exclusion of individuals with implausible EI was not justified. Given the widespread presence of misreporting in dietary surveys [24, 53], and the fact that reporting errors in the intake of individual foods and nutrients appear to correlate with reporting errors in EI to some extent at least [57, 60, 63], routine utilization of procedures to take account of EI misreporting [26] would likely improve the accuracy of studies of diet-related GHGE.

## Additional file

Additional file 1 **Table S1.** Components and scoring criteria of each of the diet quality measures used in this study. **Table S2.** Definition of food groups used in this study and estimates of GHGE. **Table S3.** Diet-related GHGE (kg CO_2_eq/d) according to categories of participants’ characteristics (*n* = 3502)**. Table S4.** Pearson correlations of diet-related GHGE and diet quality measures with EI and EI:EER. (PDF 39 kb)

## Abbreviations

BMI: Body mass index
CO_2_eq: Carbon dioxide equivalents
DASH: Dietary Approaches to Stop Hypertension
DRI: Dietary Reference Intakes
EER: Estimated energy requirement
EI: Energy intake
GHGE: Greenhouse gas emissions
HDI: Healthy diet indicator
MDS: Mediterranean diet score
MVPA: Moderate-to-vigorous physical activity
NDNS: National Diet and Nutrition Survey
PA: Physical activity

## References

[1] UK Government (2008). Climate change act.

[2] Garnett T (2011). Where are the best opportunities for reducing greenhouse gas emissions in the food system (including the food chain)?. Food Policy.

[3] Tukker A, Goldbohm RA, De Koning A, Verheijden M, Kleijn R, Wolf O (2011). Environmental impacts of changes to healthier diets in Europe. Ecol Econ.

[4] Kim B, Neff R (2009). Measurement and communication of greenhouse gas emissions from U.S. food consumption via carbon calculators. Ecol Econ.

[5] Macdiarmid JI, Kyle J, Horgan GW, Loe J, Fyfe C, Johnstone A (2012). Sustainable diets for the future: can we contribute to reducing greenhouse gas emissions by eating a healthy diet?. Am J Clin Nutr.

[6] Vieux F, Soler LG, Touazi D, Darmon N (2013). High nutritional quality is not associated with low greenhouse gas emissions in self-selected diets of French adults. Am J Clin Nutr.

[7] Temme EH, Toxopeus IB, Kramer GF, Brosens MC, Drijvers JM, Tyszler M (2015). Greenhouse gas emission of diets in the Netherlands and associations with food, energy and macronutrient intakes. Public Health Nutr.

[8] Vieux F, Darmon N, Touazi D, Soler LG (2012). Greenhouse gas emissions of self-selected individual diets in France: changing the diet structure or consuming less?. Ecol Econ.

[9] Hyland JJ, Henchion M, McCarthy M, McCarthy SN (2017). The climatic impact of food consumption in a representative sample of Irish adults and implications for food and nutrition policy. Public Health Nutr.

[10] Pan A, Sun Q, Bernstein AM, Schulze MB, Manson JE, Stampfer MJ (2012). Red meat consumption and mortality: results from 2 prospective cohort studies. Arch Intern Med.

[11] Schwingshackl L, Schwedhelm C, Hoffmann G, Lampousi AM, Knuppel S, Iqbal K (2017). Food groups and risk of all-cause mortality: a systematic review and meta-analysis of prospective studies. Am J Clin Nutr.

[12] Sinha R, Cross AJ, Graubard BI, Leitzmann MF, Schatzkin A (2009). Meat intake and mortality: a prospective study of over half a million people. Arch Intern Med.

[13] Wang X, Ouyang Y, Liu J, Zhu M, Zhao G, Bao W (2014). Fruit and vegetable consumption and mortality from all causes, cardiovascular disease, and cancer: systematic review and dose-response meta-analysis of prospective cohort studies. BMJ.

[14] Perignon M, Vieux F, Soler LG, Masset G, Darmon N (2017). Improving diet sustainability through evolution of food choices: review of epidemiological studies on the environmental impact of diets. Nutr Rev.

[15] Monsivais P, Scarborough P, Lloyd T, Mizdrak A, Luben R, Mulligan AA (2015). Greater accordance with the dietary approaches to stop hypertension dietary pattern is associated with lower diet-related greenhouse gas production but higher dietary costs in the United Kingdom. Am J Clin Nutr.

[16] Biesbroek S, Verschuren WMM, Boer JMA, van de Kamp ME, van der Schouw YT, Geelen A (2017). Does a better adherence to dietary guidelines reduce mortality risk and environmental impact in the Dutch sub-cohort of the European prospective investigation into cancer and nutrition?. Br J Nutr.

[17] Balter K, Sjors C, Sjolander A, Gardner C, Hedenus F, Tillander A (2017). Is a diet low in greenhouse gas emissions a nutritious diet? - analyses of self-selected diets in the LifeGene study. Arch Public Health.

[18] Sjors C, Hedenus F, Sjolander A, Tillander A, Balter K (2017). Adherence to dietary recommendations for Swedish adults across categories of greenhouse gas emissions from food. Public Health Nutr.

[19] Masset G, Vieux F, Verger EO, Soler LG, Touazi D, Darmon N (2014). Reducing energy intake and energy density for a sustainable diet: a study based on self-selected diets in French adults. Am J Clin Nutr.

[20] Berners-Lee M, Hoolohan C, Cammack H, Hewitt CN (2012). The relative greenhouse gas impacts of realistic dietary choices. Energy Policy.

[21] Hoolohan C, Berners-Lee M, McKinstry-West J, Hewitt CN (2013). Mitigating the greenhouse gas emissions embodied in food through realistic consumer choices. Energy Policy.

[22] Jessri M, Lou WY, L’Abbe MR (2016). Evaluation of different methods to handle misreporting in obesity research: evidence from the Canadian national nutrition survey. Br J Nutr.

[23] Murakami K, McCaffrey TA, Livingstone MB (2013). Associations of dietary glycaemic index and glycaemic load with food and nutrient intake and general and central obesity in British adults. Br J Nutr.

[24] Murakami K, Livingstone MB (2015). Prevalence and characteristics of misreporting of energy intake in US adults: NHANES 2003-2012. Br J Nutr.

[25] Mendez MA, Popkin BM, Buckland G, Schroder H, Amiano P, Barricarte A (2011). Alternative methods of accounting for underreporting and overreporting when measuring dietary intake-obesity relations. Am J Epidemiol.

[26] Banna JC, McCrory MA, Fialkowski MK, Boushey C (2017). Examining plausibility of self-reported energy intake data: considerations for method selection. Front Nutr.

[27] Murakami K, Livingstone MB (2014). Eating frequency in relation to body mass index and waist circumference in British adults. Int J Obes.

[28] Murakami K, Livingstone MB (2015). Eating frequency is positively associated with overweight and central obesity in US adults. J Nutr.

[29] Leech RM, Timperio A, Livingstone KM, Worsley A, McNaughton SA (2017). Temporal eating patterns: associations with nutrient intakes, diet quality, and measures of adiposity. Am J Clin Nutr.

[30] Public Health England and Food Standards Agency. National Diet and Nutrition Survey. Results from Years 1, 2, 3 and 4 (combined) of the Rolling Programme (2008/2009 – 2011/2012). https://www.google.co.jp/url?sa=t&rct=j&q=&esrc=s&source=web&cd=1&cad=rja&uact=8&ved=0ahUKEwjlpPTM6s3KAhXI46YKHcYTDpEQFggdMAA&url=https%3A%2F%2Fwww.gov.uk%2Fgovernment%2Fuploads%2Fsystem%2Fuploads%2Fattachment_data%2Ffile%2F310995%2FNDNS_Y1_to_4_UK_report.pdf&usg=AFQjCNGXhDQZVyuauuF80F7rFBEUKfcSEQ. Accessed 17 Oct 2017.

[31] Public Health England and Food Standards Agency. National Diet and Nutrition Survey. Results from Years 5 and 6 (combined) of the Rolling Programme (2012/2013 – 2013/2014). https://www.gov.uk/government/uploads/system/uploads/attachment_data/file/551352/NDNS_Y5_6_UK_Main_Text.pdf. Accessed 17 Oct 2017.

[32] NatCen Social Research. National Diet and Nutrition Survey Years 1–4 2008/09–2011/12. User Guide for UK Data (core & country boost data). http://doc.ukdataservice.ac.uk/doc/6533/mrdoc/pdf/6533_ndns_yrs1-4_uk_user_guide.pdf. Accessed 26 Dec 2017.

[33] NatCen Social Research. National Diet and Nutrition Survey Years 5–6 2012/13–2013/14. User Guide for UK Data. http://doc.ukdataservice.ac.uk/doc/6533/mrdoc/pdf/6533_ndns_yrs5-6_uk_user_guide.pdf. Accessed 26 Dec 2017.

[34] Rose D, Pevalin D, O’Reilly K (2005). The National Statistics Socio-Economic Classification: origins, development and use.

[35] Besson H, Brage S, Jakes RW, Ekelund U, Wareham NJ (2010). Estimating physical activity energy expenditure, sedentary time, and physical activity intensity by self-report in adults. Am J Clin Nutr.

[36] Mindell J. Appendix V to National Diet and Nutrition Survey. Results from Years 1-4 (combined) of the Rolling Programme (2008/2009-2011/12). Measuring physical activity in adults using the Recent Physical Activity Questionnaire (RPAQ). London: Public Health England. https://www.food.gov.uk/sites/default/files/ndns-appendix-v.pdf. Accessed 17 Oct 2017.

[37] Institute of Medicine (2002). Dietary reference intakes for energy, carbohydrate, fiber, fat, fatty acids, cholesterol, protein and amino acids.

[38] Brooks GA, Butte NF, Rand WM, Flatt JP, Caballero B (2004). Chronicle of the Institute of medicine physical activity recommendation: how a physical activity recommendation came to be among dietary recommendations. Am J Clin Nutr.

[39] Noel SE, Mattocks C, Emmett P, Riddoch CJ, Ness AR, Newby P (2010). Use of accelerometer data in prediction equations for capturing implausible dietary intakes in adolescents. Am J Clin Nutr.

[40] Berners-Lee M, Hoolohan C, Cammack H, Hewitt CN. Appendix A to National Diet and Nutrition Survey. Results from Years 1-4 (combined) of the Rolling Programme (2008/2009-2011/12). Dietary data collection and editing. London: Public Health England. https://www.food.gov.uk/sites/default/files/ndns-appendix-a.pdf. Accessed 17 Oct 2017.

[41] Food Standards Agency (2002). McCance and Widdowson’s the composition of foods.

[42] Huijbregts P, Feskens E, Rasanen L, Fidanza F, Nissinen A, Menotti A (1997). Dietary pattern and 20 year mortality in elderly men in Finland, Italy, and The Netherlands: longitudinal cohort study. BMJ.

[43] Struijk EA, Beulens JW, May AM, Fransen HP, Boer JM, de Wit GA (2014). Dietary patterns in relation to disease burden expressed in disability-adjusted life years. Am J Clin Nutr.

[44] Trichopoulou A, Orfanos P, Norat T, Bueno-de-Mesquita B, Ocke MC, Peeters PH (2005). Modified Mediterranean diet and survival: EPIC-elderly prospective cohort study. BMJ.

[45] Fung TT, Chiuve SE, McCullough ML, Rexrode KM, Logroscino G, Hu FB (2008). Adherence to a DASH-style diet and risk of coronary heart disease and stroke in women. Arch Intern Med.

[46] Penney TL, Jones NRV, Adams J, Maguire ER, Burgoine T, Monsivais P (2017). Utilization of away-from-home food establishments, dietary approaches to stop hypertension dietary pattern, and obesity. Am J Prev Med.

[47] Reedy J, Krebs-Smith SM, Miller PE, Liese AD, Kahle LL, Park Y (2014). Higher diet quality is associated with decreased risk of all-cause, cardiovascular disease, and cancer mortality among older adults. J Nutr.

[48] Jankovic N, Geelen A, Streppel MT, de Groot LC, Orfanos P, van den Hooven EH (2014). Adherence to a healthy diet according to the World Health Organization guidelines and all-cause mortality in elderly adults from Europe and the United States. Am J Epidemiol.

[49] Green R, Milner J, Dangour AD, Haines A, Chalabi Z, Markandya A (2015). The potential to reduce greenhouse gas emissions in the UK through healthy and realistic dietary change. Clim Chang.

[50] Huang TT, Roberts SB, Howarth NC, McCrory MA (2005). Effect of screening out implausible energy intake reports on relationships between diet and BMI. Obes Res.

[51] Black AE (2000). Critical evaluation of energy intake using the Goldberg cut-off for energy intake:basal metabolic rate. A practical guide to its calculation, use and limitations. Int J Obes Relat Metab Disord.

[52] NatCen Social Research. National Diet and Nutrition Survey Years 1–6, 2008/09–2013/14. Combining Years 1–4 and Years 5 and 6 data - Advice for users. https://www.google.co.jp/url?sa=t&rct=j&q=&esrc=s&source=web&cd=1&cad=rja&uact=8&ved=0ahUKEwjm8-bEwJXZAhVGWbwKHZXpCXwQFggnMAA&url=http%3A%2F%2Fdoc.ukdataservice.ac.uk%2Fdoc%2F6533%2Fmrdoc%2Fpdf%2F6533_combining_the_y1-4_and_y56_datasets_on_the_archive_final_version.pdf&usg=AOvVaw36XsNPUN6ZDVHgZkbrdu0o. Accessed 8 Feb 2018.

[53] Livingstone MB, Black AE (2003). Markers of the validity of reported energy intake. J Nutr.

[54] Seves SM, Verkaik-Kloosterman J, Biesbroek S, Temme EH (2017). Are more environmentally sustainable diets with less meat and dairy nutritionally adequate?. Public Health Nutr.

[55] Perignon M, Masset G, Ferrari G, Barré T, Vieux F, Maillot M (2016). How low can dietary greenhouse gas emissions be reduced without impairing nutritional adequacy, affordability and acceptability of the diet? A modelling study to guide sustainable food choices. Public Health Nutr.

[56] Derbyshire E (2017). Associations between red meat intakes and the micronutrient intake and status of UK females: a secondary analysis of the UK National Diet and nutrition survey. Nutrients.

[57] Subar AF, Kipnis V, Troiano RP, Midthune D, Schoeller DA, Bingham S (2003). Using intake biomarkers to evaluate the extent of dietary misreporting in a large sample of adults: the OPEN study. Am J Epidemiol.

[58] Rosell MS, Hellenius MLB, De Faire UH, Johansson GK (2003). Associations between diet and the metabolic syndrome vary with the validity of dietary intake data. Am J Clin Nutr.

[59] Heitmann BL, Lissner L (1995). Dietary underreporting by obese individuals: is it specific or non-specific?. BMJ.

[60] Murakami K, Sasaki S, Uenishi K (2012). The degree of misreporting of the energy-adjusted intake of protein, potassium, and sodium does not differ among under-, acceptable, and over-reporters of energy intake. Nutr Res.

[61] Nielsen BM, Nielsen MM, Toubro S, Pedersen O, Astrup A, Sorensen TI (2009). Past and current body size affect validity of reported energy intake among middle-aged Danish men. J Nutr.

[62] Black AE (2000). The sensitivity and specificity of the Goldberg cut-off for EI:BMR for identifying diet reports of poor validity. Eur J Clin Nutr.

[63] Freedman LS, Commins JM, Moler JE, Willett W, Tinker LF, Subar AF (2015). Pooled results from 5 validation studies of dietary self-report instruments using recovery biomarkers for potassium and sodium intake. Am J Epidemiol.

[64] Bertoluci G, Masset G, Gomy C, Mottet J, Darmon N (2016). How to build a standardized country-specific environmental food database for nutritional epidemiology studies. PLoS One.

[65] Aleksandrowicz L, Green R, Joy EJ, Smith P, Haines A (2016). The impacts of dietary change on greenhouse gas emissions, land use, water use, and health: a systematic review. PLoS One.

[66] Food Standards Agency. NDNS Previous Survey Reports. http://webarchive.nationalarchives.gov.uk/20100406130654/food.gov.uk/science/dietarysurveys/ndnsdocuments/ndnsprevioussurveyreports/. Accessed 17 Oct 2017.

