# Supplementary material for: Greenhouse gas emissions of self-selected diets in the UK and their association with diet quality: is energy under-reporting a problem?
Source: Nutr J. 2018 Feb 21;17:27. doi: 10.1186/s12937-018-0338-x (PMC5822528; doi:10.1186/s12937-018-0338-x)
Supplement: Supplementary file 1 — Table S1. Components and scoring criteria of each of the diet quality measures used in this study. Table S2. Definition of food groups used in this study and estimates of GHGE. Table S3. Diet-related GHGE (kg CO2eq/d) according to categories of participants’ characteristics (n = 3502). Table S4. Pearson correlations of diet-related GHGE and diet quality measures with EI and EI:EER. (PDF 39 kb) [file 12937_2018_338_MOESM1_ESM.pdf]

Table S1. Components and scoring criteria of each of the diet quality measures used in this study

	Maximum score	Minimum score
Healthy diet indicator*	1 point	0 point
Saturated fat (% of energy)	<10	≥10
Polyunsaturated fat (% of energy)	6 to 10	<6 or >10
Cholesterol (mg/d)	<300	≥300
Protein (% of energy)	10 to 15	<10 or >15
Dietary fibre (g/d)	≥25	25
Fruits and vegetables (g/d)	≥400	<400
Non-milk extrinsic sugar (% of energy)	<10	≥10
Mediterranean diet score†	1 point	0 point
Vegetables (g/d)	Above or equal to sex-specific median	Below sex-specific median
Legumes (g/d)‡	Above or equal to sex-specific median	Below sex-specific median
Fruits, nuts and seeds (g/d)	Above or equal to sex-specific median	Below sex-specific median
Cereals (g/d)	Above or equal to sex-specific median	Below sex-specific median
Fish (g/d)	Above or equal to sex-specific median	Below sex-specific median
Unsaturated: saturated fat ratio	Above or equal to sex-specific median	Below sex-specific median
Meat (g/d)	Below or equal to sex-specific median	Above sex-specific median
Dairy products (g/d)	Below or equal to sex-specific median	Above sex-specific median
Alcohol (g/d)	Men: 10-50; women: 5-25	Men: <10 or >50; women: <5 or >25
DASH score§	5 points	1 point
Fruits (including fruit juice) (g/d)	Sex-specific quintile 5	Sex-specific quintile 1
Vegetables (g/d)	Sex-specific quintile 5	Sex-specific quintile 1
Nuts and legumes (g/d)||	Sex-specific quintile 5	Sex-specific quintile 1
Whole grain foods (g/d)¶	Sex-specific quintile 5	Sex-specific quintile 1
Low fat dairy products (g/d)**	Sex-specific quintile 5	Sex-specific quintile 1
Red and processed meats (g/d)††	Sex-specific quintile 1	Sex-specific quintile 5
Non-milk extrinsic sugar (g/d)	Sex-specific quintile 1	Sex-specific quintile 5
Sodium (mg/d)	Sex-specific quintile 1	Sex-specific quintile 5

DASH, Dietary approaches to Stop Hypertension.

* Possible score ranging from 0 to 7.

† Possible score ranging from 0 to 9.

‡ Including "beans and pulses".

§ Scoring of the components of the DASH score depends on the sex-specific quintile (1, 2, 3, 4 or 5 points). Possible score ranging from 8 to 40.

|| Including "nuts and seeds" and "beans and pulses".

¶ Including wholemeal bread (sub food group code in the National Diet and Nutrition Survey rolling programme: 3R), brown, granary and wheatgerm bread (sub food group code: 59R), high fibre breakfast cereals (sub food group code: 5R), whole grain pasta (food code in the National Diet and Nutrition Survey rolling programme: 35 and 36) and brown rice (food code: 48-53).

** Including yogurt (sub food group code: 15B), cottage cheese (sub food group code: 14A), skimmed milk (sub food group code: 12R), 1%fat milk (sub food group code: 60R) and semi-skimmed milk (sub food group code: 11R).

†† Including "red meat", manufactured coated chicken/turkey products (sub food group code: 26A) and manufactured chicken products (including ready meals) (sub food group code: 27A).

Table S2. Definition of food groups used in this study and estimates of GHGE

Food group used in this study	Sub food group in NDNS rolling programme		GHGE (kg CO_2eq per kg consumed)
	Code	Description	
Red meat	22A	Ready meals/meal centres based on bacon and ham	10.1
	22B	Other bacon and ham (including homemade dishes)	10.1
	23A	Manufactured beef products (including ready meals)	9.8
	23B	Other beef & veal (including homemade recipe dishes)	35.4
	24A	Manufactured lamb products (including ready meals)	32.5
	24B	Other lamb (including homemade recipe dishes)	32.5
	25A	Manufactured pork products (including ready meals)	10.1
	25B	Other pork (including homemade recipe dishes)	9
	28R	Liver and dishes	33.9
	29R	Burgers and kebabs purchased	9.8
	30A	Ready meals based on sausages	10.1
	30B	Other sausages (including homemade dishes)	10.1
	31A	Meat pies and pastries (manufactured)	9.8
	31B	Meat pies and pastries (homemade)	9.8
	32A	Other meat products (manufactured including ready meals)	9.8
	32B	Other meat (including homemade recipe dishes)	33.9
White meat	26A	Manufactured coated chicken/turkey products	6.2
	27A	Manufactured chicken products (including ready meals)	6.2
	27B	Other chicken/turkey (including homemade recipe dishes)	7.6
Fish	33R	White fish coated or fried	10.6
	34C	Manufactured white fish products (including ready meals)	10.6
	34D	Other white fish (including homemade dishes)	10.6
	34E	Manufactured shellfish products (including ready meals)	19.9
	34F	Other shellfish (including homemade dishes)	19.9
	34G	Manufactured canned tuna products (including ready meals)	5.9
	34H	Other canned tuna (including homemade dishes)	5.9
	35A	Manufactured oily fish products (including ready meals)	10.6
	35B	Other oily fish (including homemade dishes)	10.6
Dairy products	10R	Whole milk	2
	11R	Semi-skimmed milk	2
	60R	1% Milk	2
	12R	Skimmed milk	2
	13A	Infant formula	2
	13B	Cream (including imitation cream)	2
	13R	Other milk	2
	14A	Cottage cheese	18.3
	14B	Cheddar cheese	18.3
	14R	Other cheese	18.3
	15B	Yogurt	2
	15C	Fromage frais and other dairy desserts (manufactured)	2
	15D	Dairy desserts (homemade)	2
	53R	Ice cream	5
Eggs	16C	Manufactured egg products including ready meals	4.7
	16D	Other eggs and egg dishes including homemade	4.7

Table S2. Continued

Food group used in this study	Sub food group in NDNS rolling programme		GHGE (kg CO_2eq per kg consumed)
	Code	Description	
Cereals	1C	Pizza	5.6
	1D	Pasta (manufactured products and ready meals)	1
	1E	Pasta (other, including homemade dishes)	1
	1F	Rice (manufactured products and ready meals)	2.5
	1G	Rice (other, including homemade dishes)	2.5
	1R	Other cereals	1.7
	2R	White bread (not high fibre, not multiseed bread)	1.7
	3R	Wholemeal bread	1.7
	59R	Brown, granary and wheatgerm bread	1.7
	4R	Other bread	1.7
	5R	High fibre breakfast cereals	1.7
	6R	Other breakfast cereals (not high fibre)	1.7
Potatoes	38A	Chips purchased including takeaway	2.3
	38C	Other manufactured potato products fried/baked	2.3
	38D	Other fried/roast potatoes (including homemade dishes)	1.5
	39A	Other potato products and dishes (manufactured)	1.5
	39B	Other potatoes (including homemade dishes)	1.5
Vegetables	36A	Carrots (raw)	2
	36B	Salad and other raw vegetables	2
	36C	Tomatoes raw	2
	37A	Peas not raw	2
	37D	Leafy green vegetables not raw	2
	37E	Carrots not raw	2
	37F	Tomatoes not raw	2
	37K	Meat alternatives (including ready meals and homemade dishes)	2
	37L	Other manufactured vegetable products (including ready meals)	2
	37M	Other vegetables (including homemade dishes)	2
Beans and pulses	37B	Green beans not raw	1.5
	37C	Baked beans	1.5
	37I	Beans and pulses (including ready meal & homemade dishes)	1.5
Nuts and seeds	56R	Nuts and seeds	2.1
Fruit	40A	Apples and pears not canned	1.4
	40B	Citrus fruit not canned	1.4
	40C	Bananas	1.4
	40D	Canned fruit in juice	1.9
	40E	Canned fruit in syrup	1.9
	40R	Other fruit not canned	1.4

Table S2. Continued

Food group used in this study	Sub food group in NDNS rolling programme		GHGE (kg CO_2eq per kg consumed)
	Code	Description	
Fats and oils	17R	Butter	31.2
	18A	Polyunsaturated margarine	20.9
	18B	Polyunsaturated oils	2.7
	19A	Polyunsaturated low fat spread	20.9
	19R	Low fat spread not polyunsaturated	20.9
	20A	Block margarine	20.9
	20B	Soft margarine not polyunsaturated	20.9
	20C	Other cooking fats and oils not polyunsaturated	2.7
	21A	Reduced fat spread (polyunsaturated)	20.9
	21B	Reduced fat spread (not polyunsaturated)	20.9
Sugar and confectioneries	7A	Biscuits (manufactured/retail)	2.4
	7B	Biscuits (homemade)	2.4
	8B	Fruit pies (manufactured)	3.2
	8C	Fruit pies (homemade)	3.2
	8D	Buns cakes and pastries (manufactured)	2.6
	8E	Buns cakes and pastries (homemade)	2.6
	9C	Cereal based milk puddings (manufactured)	3.2
	9D	Cereal based milk puddings (homemade)	3.2
	9E	Sponge puddings (manufactured)	3.2
	9F	Sponge puddings (homemade)	3.2
	9G	Other cereal based puddings (manufactured)	3.2
	9H	Other cereal based puddings (homemade)	3.2
	41A	Sugar	1.7
	41B	Preserves	3.5
	41R	Sweet spreads fillings and icing	1.7
	43R	Sugar confectionery	2.9
	44R	Chocolate confectionery	2.9
	42R	Crisps and savoury snacks	4.6
	50C	Soup (manufactured/retail)	4.6
	50D	Soup (homemade)	4.6
Soft drinks	57A	Soft drinks not low calorie concentrated	2
	57B	Soft drinks not low calorie carbonated	2
	57C	Soft drinks not low calorie, ready to drink, still	2
	58A	Soft drinks low calorie concentrated	2
	58B	Soft drinks low calorie carbonated	2
	58C	Soft drinks low calorie, ready to drink, still	2
Fruit juice	45R	Fruit juice	2.4
	61R	Smoothies	2.4

Table S2. Continued

Food group used in this study	Sub food group in NDNS rolling programme		GHGE (kg CO_2eq per kg consumed)
	Code	Description	
Alcoholic beverages	47A	Liqueurs	1.5
	47B	Spirits	1.5
	48A	Wine	1.5
	48B	Fortified wine	1.5
	48C	Low alcohol and alcohol free wine	1.5
	49A	Beers and lagers	1.5
	49B	Low alcohol & alcohol free beer & lager	1.5
	49C	Cider and perry	1.5
	49D	Low alcohol & alcohol free cider & perry	1.5
	49E	Alcoholic soft drinks (Alcopops)	1.5
Tea, coffee and water	51A	Coffee (made up weight)	0.6
	51B	Tea (made up)	0.03
	51C	Herbal tea (made up)	0.03
	51D	Bottled water still or carbonated	0.5

CO_2eq, carbon dioxide equivalents; GHGE, greenhouse gas emissions; NDNS, National Diet and Nutrition Survey.

GHGE values of each food group were taken from Green et al. [49]. The following four sub food groups appearing in NDNS rolling programme were not considered in the calculation of diet-related GHGE as well as of food group intake: tap water only (51R), beverages dry weight (50A), nutrition powders and drinks (50E), and savoury sauces pickles gravies & condiments (50R).

Table S3. Diet-related GHGE (kg CO_2eq/d) according to categories of participants' characteristics (*n* = 3502)

	n	Mean	SD
Age category			
19-34 y	812	5.9[a]	2.6
35-49 y	1068	5.7[b,c]	2.1
50-64 y	865	5.8[a,b]	2.0
≥65 y	757	5.5[c,d]	1.7
P *		<0.0001	
Sex			
Male	1429	6.5	2.4
Female	2073	5.0	1.5
P *		<0.0001	
Ethnicity			
White	3270	5.8	2.0
Nonwhite	232	4.9	2.6
P *		<0.0001	
Socioeconomic classification			
Higher and managerial occupation	1409	5.8[a]	2.0
Intermediate occupation	721	5.8[a]	2.1
Routine and manual occupation	1219	5.6[a]	2.3
Other	153	5.4[a]	1.9
P *		0.004	
Smoking status			
Current	830	5.9[a]	2.3
Former	833	5.8[a]	2.0
Never	1839	5.6[b]	2.1
P *		0.001	
Physical activity (%)			
Sedentary	1499	5.3[a]	1.9
Low active	678	5.7[b]	2.0
Active	628	5.9[b]	2.1
Very active	697	6.4[c]	2.4
P *		<0.0001	

CO_2eq, carbon dioxide equivalents; GHGE, greenhouse gas emissions.

* On the basis of the independent *t* test for sex and ethnicity and ANOVA for other variables. When the overall *P* from ANOVA was <0.05, a Bonferroni post hoc test was performed; values within each variable with different superscript letters are significantly different (*P* <0.05).

Table S4. Pearson correlations of diet-related GHGE and diet quality measures with EI and EI:EER*

	All (*n* = 3502)†				Plausible reporters (*n* = 1895)				Under-reporters (*n* = 1578)			
	EI (kJ/d)		EI:EER		EI (kJ/d)		EI:EER		EI (kJ/d)		EI:EER	
	r	*P*	*r*	*P*	*r*	*P*	*r*	*P*	*r*	*P*	*r*	*P*
GHGE (kg CO_2eq/d)	0.72	<0.0001	0.40	<0.0001	0.67	<0.0001	0.18	<0.0001	0.68	<0.0001	0.35	<0.0001
HDI	-0.10	<0.0001	-0.08	<0.0001	-0.04	0.06	0.007	0.76	-0.12	<0.0001	-0.09	0.0005
MDS	0.08	<0.0001	0.07	0.0007	0.04	0.052	-0.01	0.62	0.08	0.001	0.11	<0.0001
DASH score	-0.17	<0.0001	-0.12	<0.0001	-0.14	<0.0001	-0.09	0.0001	-0.13	<0.0001	-0.05	0.04

CO_2eq, carbon dioxide equivalents; DASH, Dietary Approaches to Stop Hypertension; EER, estimated energy requirement; EI, energy intake; GHGE, greenhouse gas emissions; HDI, healthy diet indicator; MDS, Mediterranean diet score.

* Plausible reporters were defined as participants with an EI:EER 0.70-1.43; under-reporters were defined as participants with an EI:EER <0.70.

† Including over-reporters (*n* = 29), defined as participants with an EI:EER >1.43.
